# Sex-dependent effect of sublethal copper concentrations on de novo cholesterol synthesis in astrocytes and their possible links to variations in cholesterol and amyloid precursor protein levels in neuronal membranes

**DOI:** 10.1186/s13293-023-00578-9

**Published:** 2024-01-08

**Authors:** Marlene Zubillaga, Julia Tau, Diana Rosa, M. José Bellini, Nathalie Arnal

**Affiliations:** 1 Laboratorio de Neurociencia, Instituto de Investigaciones Bioquímicas de La Plata (INIBIOLP), Consejo Nacional de Investigaciones Científicas y Técnicas (CONICET), Universidad Nacional de La Plata (UNLP), Calle 60 y 120, CP 1900 La Plata, Argentina; 2 Instituto de Investigaciones Bioquímicas de La Plata (INIBIOLP), Consejo Nacional de Investigaciones Científicas y Técnicas (CONICET), Universidad Nacional de La Plata (UNLP), Calle 60 y 120, CP 1900 La Plata, Argentina; 3 Laboratorio de Nutrición Mineral, Fac. Cs Veterinarias, UNLP (Universidad Nacional de La Plata), Calle 60, CP 1900 La Plata, Argentina; 4 Laboratorio de Neurobiología y Cognición en el Envejecimiento y Enfermedades Neurodegenerativas, Instituto de Investigaciones Bioquímicas de La Plata (INIBIOLP), Consejo Nacional de Investigaciones Científicas y Técnicas (CONICET), Universidad Nacional de La Plata (UNLP), Calle 60 y 120, CP 1900 La Plata, Argentina

**Keywords:** Cholesterol, Copper, Sexual differences, Astrocytes, Neurons

## Abstract

**Background:**

Cholesterol (Cho) is an essential lipophilic molecule in cells; however, both its decrease and its increase may favor the development of neurological diseases such as Alzheimer’s disease (AD). Although copper (Cu) is an essential trace metal for cells, the increased plasma concentration of its free form has been linked with AD development and severity. AD affects aged people, but its prevalence and severity are higher in women than in men. We have previously shown that Cu promotes Cho de novo synthesis in immature neurons as well as increased Cho in membrane rafts and Aβ levels in culture medium, but there are no results yet regarding sex differences in the effects of sublethal Cu exposure on Cho de novo synthesis.

**Methods:**

We examined the potential sex-specific impact of sublethal Cu concentrations on de novo Cho synthesis in primary cultures of male and female astrocytes. We also explored whether this had any correlation with variations in Cho and APP levels within neuronal membrane rafts.

**Results:**

Flow cytometry analysis demonstrated that Cu treatment leads to a greater increase in ROS levels in female astrocytes than in males. Furthermore, through RT-PCR analysis, we observed an upregulation of SREBP-2 and HMGCR. Consistently, we observed an increase in de novo Cho synthesis. Finally, western blot analysis indicated that the levels of ABCA1 increase after Cu treatment, accompanied by a higher release of radiolabeled Cho and an elevation in Cho and APP levels in neuronal membrane rafts. Importantly, all these results were significantly more pronounced in female astrocytes than in males.

**Conclusions:**

Our findings confirm that Cu stimulates Cho synthesis in astrocytes, both in a ROS-dependent and -independent manner. Moreover, female astrocytes displayed elevated levels of HMGCR, and de novo Cho synthesis compared to males following TBH and Cu treatments. This corresponds with higher levels of Cho released into the culture medium and a more significant Cho and APP rise within neuronal rafts. We consider that the increased risk of AD in females partly arises from sex-specific responses to metals and/or exogenous substances, impacting key enzyme regulation in various biochemical pathways, including HMGCR.

## Highlights

- Cho and Cu overload are both linked with AD development and severity.
- AD prevalence and severity are higher in women than in men.
- We showed for the first time that female astrocytes seem to be more susceptible to ROS-inducing substances than males and that both ROS and Cu favor Cho de novo synthesis more in females than in male astrocytes.
- We found higher Cho and APP levels in females than in male neuronal rafts, matching the higher release of Cho to the culture medium in female astrocytes after Cu treatment.

## Plain language summary

Alzheimer’s disease (AD) primarily affects the elderly and is linked to excess cholesterol (Cho) and copper (Cu). It is more prevalent and severe in women. Previous research suggested that Cu may enhance Cho synthesis in developing neurons, raising Cho levels in specialized membrane structures (rafts) and Aβ protein in the culture medium. However, the specific effects of Cu exposure on Cho synthesis in males and females are not entirely understood. We conducted experiments using astrocytes, the primary cells in the brain that produce Cho in adults, and neurons, both from male and female rats. We exposed them to non-lethal levels of Cu to explore its potential sex-related effects on (1) Cho metabolism in astrocytes, and (2) The relationship between the Cho released by astrocytes and the levels of Cho and amyloid precursor protein (APP) in neuronal membrane rafts. Our findings suggested that reactive oxygen species (ROS)-responsive sensitivity is higher in females than in male astrocytes. Cu, alongside ROS, promoted Cho synthesis, with female astrocytes being more susceptible. These released more Cho into the medium after Cu exposure, and Cho and APP levels were also higher in female neuronal rafts exposed to Cu-treated astrocyte-conditioned medium. Our results thus imply that the higher risk of AD in females may arise partly from sex-related disparities in cellular responses to external substances, impacting such crucial biochemical pathways as Cho synthesis.

## Background

Cholesterol (Cho) is one of the main structural lipids of cell membranes, but also a precursor of steroid hormones, fats, and lipophilic vitamins, among others. Given that peripheral Cho cannot pass the blood–brain barrier, brain cells should be able to meet its Cho requirements. Several reports show that neurons and astrocytes in the immature brain are responsible for synthesizing their own Cho [1, 2]. In the mature brain, however, neurons acquire Cho primarily from astrocytes, which are responsible for its synthesis and secretion [2–4]. These astrocytes make up at least 30% of the brain’s cell population. Their roles include providing structural and metabolic support to neurons by secreting most of the synthesized Cho, retaining only the Cho necessary for their proper function. It is important to note that the balance of Cho is carefully controlled in both mature and immature brains. Any significant deviations, whether increasing or decreasing this lipid, may contribute to the onset of neurological disorders such as depression and Alzheimer’s disease (AD) [5].

AD is a multifactorial neurodegenerative disease in which hypercholesterolemia and copper (Cu) overload are considered potential risk factors. Numerous studies demonstrate that changes in Cho levels within membrane rafts (lipid-rich microdomains containing Cho and sphingolipids) can stimulate the amyloidogenic processing of the amyloid precursor protein (APP). This, in turn, leads to elevated beta-amyloid peptide (Aβ) levels, which may play a role in the onset and advance of AD [6, 7].

Cu is an essential transition metal for proper cell function, serving as a cofactor for numerous enzymes. Several studies demonstrate that an excessive amount of its free form is linked to the initiation and advance of AD [8–10]. The possible sources of the excess of free Cu in AD are not fully understood. Brewer suggested that the use of Cu pipes for the supply of drinking water may have caused the epidemic increase of AD in developing countries [11]. In addition, the use of Cu-based pesticides and Cu intrauterine devices may also be a cause of Cu overload in humans [12, 13]. Excess Cu not only increases oxidative stress through the Harber-Weiss or Fenton reactions by modifying molecular oxygen leading to the production of ROS [14, 15] but also contributes to Cho de novo synthesis, both in a manner influenced by reactive oxygen species (ROS) and independently of ROS [16].

Numerous reviews and meta-analyses consistently demonstrate a higher prevalence of AD in women than in men [17–19]. Additionally, several studies provide insights into the relationship between greater hippocampal atrophy, cognitive decline, and elevated levels of Aβ42 and hyperphosphorylated Tau (two key markers of AD) in women, and to a lesser extent in men [20]. Given that estrogens play a protective role in the brain, the onset of menopause in middle-aged women, which leads to a decline in estrogen levels, has been proposed as a contributing factor to the higher prevalence of AD in women [18, 21]. However, until now the most significant risk factor for AD development is the longer average lifespan of women than men [17]. Additionally, previous studies have identified sex-based differences in the genetic predictors of AD biomarkers [22].

Our primary objective was thus to explore the potential sex-dependent impact of sublethal Cu concentrations on de novo Cho synthesis in primary cultures of female and male astrocytes. Given that these cells play a crucial role in synthesizing and delivering Cho to neurons in the mature brain, we focused on the following aspects: (1) Generation of ROS, superoxide dismutase (SOD) activity, and lipid peroxidation (measured as TBARS); (2) De novo Cho synthesis, along with the expression of key molecules such as the sterol regulatory element-binding protein 2 (SREBP-2) and 3-hydroxy-3-methylglutaryl-coenzyme-A reductase (HMGCR); (3) Levels of ATP-binding cassette transporter A1 (ABCA1) involved in Cho efflux, and the release of labeled Cho from astrocytes into the culture medium. A second objective was to determine whether there is a relationship between the levels of Cho released by astrocytes and the levels of Cho and APP in neuronal membrane rafts. To investigate this, we exposed primary cultures of neurons from both female and male subjects to a conditioned medium obtained from astrocytes exposed to Cu (or a control medium).

## Materials and methods

Sodium acetate^[14C]^ (56.8 Ci/mol) was purchased from Perkin Elmer (Boston, MA, USA), and 1,1,3,3-tetramethoxypropane (TMP), and resazurin sodium salt (AlamarBlue^®^) from Sigma-Aldrich (St. Louis, Missouri, US). All other chemicals used were of analytical grade and were purchased from Merck (Darmstadt, Germany), Natocor (Córdoba, Argentina), or Carlo Erba (Milan, Italy).

### Cell culture

Astrocyte primary cultures were prepared by mechanical dissociation of the cerebral cortex and hippocampus from newborn Sprague–Dawley rats, as previously reported [23]. Briefly, P0–P2 male and female pups, distinguished by a larger genital papilla and longer anogenital distance in male than in female pups, were anesthetized by hypothermia followed by decapitation with sharp surgical scissors. The cerebral cortex and hippocampus were isolated under a dissecting microscope and cleaned of choroid plexus and meninges. Cell suspensions were obtained by repeatedly pipetting the cortical tissue with Pasteur pipettes of different sizes. The cell suspension was centrifuged at 300 × g for 5 min to remove detritus, and cells were counted in a Neubauer chamber by trypan blue staining. After centrifugation, cells were placed in 75 cm^3^ flasks coated with poly-l-lysine and DMEM/F12 (1:1) (Emeve medium, MicroVet SRL laboratory, Argentina), supplemented with 10% fetal bovine serum (FBS) (Natocor, Cordoba, Argentina) and penicillin–streptomycin (1X) (Laboratorio Serendipia, Colombia), and were maintained in a humidified incubator at 37 °C with 5% CO_2_. Half of the medium was changed every 3 days. When cells reached 90% confluence, enriched astrocyte cultures were obtained after overnight shaking in a tabletop shaker (Thermo Forma, Marietta, Ohio, USA) in an incubator at 37 °C with 5% CO_2_ to minimize oligodendrocyte and microglia contamination. The astrocytes were then seeded onto fresh P100 plates and incubated for 2–3 days before use.

Neuronal primary cultures were prepared from the brains of 0 to 2-day-old Sprague–Dawley pups using established procedures [24] with minor modifications. Briefly, brains were removed aseptically, placed in DMEM/F12 (1:1) (Emeve, MicroVet SRL laboratory, Argentina), and the blood vessels and meninges were carefully removed under a dissecting microscope. Brain cortexes were isolated and dissociated by digestion with a solution of 0.05% trypsin (Sigma) containing DNase I (0.06%) (Sigma) for 10 min at 37 °C. The digestion reaction was stopped with DMEM/F12 medium containing 10% FBS (Natocor, Córdoba Argentina). The cells were pelleted by centrifugation at 200 g for 5 min and resuspended in DMEM/F12 supplemented with 10% FBS (Natocor, Córdoba Argentina), glucose 0.25% (Sigma-Aldrich^®^), vitamin mix 1% (Microvet, Argentina), glutamine 2 mM (Sigma-Aldrich^®^) and penicillin–streptomycin (1X). The cell suspension was plated, at a density of 3000 cells/mm^2^, onto 12 mm glass coverslips coated with poly-d-lysine (Sigma). The neuron cultures were kept at 37 °C in a humidified incubator with 5% CO_2_ for 10 to 14 days.

### Cell viability

To determine the highest Cu concentrations that did not negatively impact cell viability (sublethal concentration), we exposed astrocytes to varying concentrations of CuSO_4_ (ranging from 0 to 1200 µM) for 24 h. After this incubation period, we assessed cell viability using the resazurin method [25]. This technique relies on the reduction of resazurin by viable cells, resulting in the production of a fluorescent product known as resorufin. Specifically, treated cells were incubated with 10 µL of AlamarBlue^®^ (Thermo Fisher Scientific) at a concentration of 0.11 mg/mL in 100 µL of phenol red-free DMEM/F12 at 37 °C for 2 h. Cell viability was subsequently determined using fluorescence spectrophotometry, with excitation and emission wavelengths set at 535 nm and 595 nm, respectively, employing a Beckman Coulter DTX 880 plate reader.

### Astrocyte and neuronal treatments

To perform the various techniques, astrocytes were cultured, using different well sizes or Petri dishes as needed, until they reached a semi-confluent state, and were subsequently treated with CuSO_4_ for 24 h. To assess ROS levels, SOD activity, lipid oxidation, and mRNA expression via RT-qPCR, astrocytes were seeded in 24-well plates, and, to evaluate de novo Cho synthesis, they were cultured in 6-well plates. Finally, for both Western blot analysis and to determine total Cho levels, astrocytes were seeded in P100 Petri dishes. After 24 h treatment, the culture media (conditioned media) were used for experiments with neurons, while the astrocytes were processed following the protocols described for each technique.

Neurons were cultured at a density of 200,000 cells per well in 48-well plates with glass coverslips treated with poly-D-lysine. The culture media were refreshed every 3 days, and on the tenth day, neurons were treated for 24 h with 500 µL of the astrocyte-conditioned medium, obtained as previously outlined. Subsequently, neurons were processed according to the procedures detailed in the “Cho in Lipid Rafts” and “Western Blotting” methodologies.

### Determination of ROS

Astrocytes were washed with phosphate-buffered saline (PBS) and harvested with 0.25% p/v trypsin. The trypsin reaction was halted with DMEM/F12 medium containing 10% FBS (Natocor, Córdoba Argentina). Cells were then centrifuged at 200 g for 5 min, resuspended in FBS-free DMEM/F12, and centrifuged again at 4000 g for 5 min. Subsequently, the cell pellets were resuspended in a medium without FBS and with 2ʹ-7ʹ-dichlorodihydrofluorescein diacetate (DCF-DA) (Invitrogen) at a concentration of 10 mM for 60 min at 37 °C. As a positive control for ROS generation, cells were treated with tert-butyl-hydroperoxide (TBH) from Sigma-Aldrich at a concentration of 500 μM for 60 min at 37 °C. Additionally, a negative control for ROS production was included, using 30 µM N-acetylcysteine (NAC) (Sigma), as previously described [26, 27]. The fluorescence emitted was quantified using flow cytometry (Accuri C6 Plus, BD).

### SOD activity

SOD activity was measured in astrocyte homogenates using the method described by Misra and Fridovich [28]. Briefly, following the treatment, astrocytes were detached using a 0.25% [w/v] trypsin solution (Sigma). The trypsinization process was halted by adding DMEM/F12 supplemented with FBS, and the cells were resuspended in PBS. Each sample received 1X lysis buffer (20 mM HEPES pH 7.40, 100 mM NaCl, 5 mM EDTA, 1% Triton X-100, supplemented with 380 µL of protease and phosphatase inhibitor cocktail from Thermo Scientific -78442). These samples were then centrifuged at 10,000 rpm for 15 min at 4 °C, and the resulting supernatant was employed to determine SOD activity. In this method, the autoxidation of epinephrine to adrenochrome occurs under alkaline conditions. SOD competes with this reaction, thereby reducing the rate of adrenochrome formation. To determine the SOD concentration in the samples, an aliquot of cell homogenate was combined with 970 µL of reaction buffer (62.5 mM Na_2_CO_3_/NaHCO_3_, pH 10.2, with 125 µM EDTA) and 20 µL of epinephrine (10.5–11.0 mg epinephrine in 2 mL of 0.1 N acetic acid). The reduction rate of adrenochrome formation was then measured over 2 min using an Agilent 8453 UV–visible spectrophotometer. In addition, a blank reaction without homogenate was conducted to establish the rate of epinephrine autoxidation (referred to as DRef). An enzymatic unit (U—units of inhibition of optical density) was defined as the quantity that inhibits 50% of autoxidation. The enzyme’s activity was calculated in terms of U per minute per µg of total cellular protein.

### Thiobarbituric acid-reactive substances (TBARS)

Astrocytes were detached using a 0.25% [w/v] trypsin solution (Sigma). The trypsinization process was halted by adding DMEM/F12 supplemented with FBS, and the cells were resuspended in PBS. Each sample received 1X lysis buffer (20 mM HEPES pH 7.40, 100 mM NaCl, 5 mM EDTA, 1% Triton X-100, and supplemented with 380 µL of protease and phosphatase inhibitor cocktail from Thermo Scientific -78,442). These samples were then centrifuged at 10,000 rpm for 15 min at 4 °C, and the resulting supernatant was employed to determine the extent of lipid peroxidation in astrocytes by analyzing the levels of TBARS as described by Yagi [29]. Briefly, 500 μL of 0.8% thiobarbituric acid (TBA) and 500 μL of acetic acid (10% pH 3.5) were added to 67 μL of cell homogenate. The sample was heated at 95 °C for 60 min. The absorbance was measured on a Beckman Coulter DTX 880 plate reader at 532 nm. The chromophore concentration was determined using a calibration curve, constructed using fresh 1,1,3,3-tetramethoxypropane (TMP) solutions (Sigma-Aldrich, Argentina). Lipid peroxidation levels were calculated in terms of µmol of TBARS per µg of total cellular protein.

### De novo Cho synthesis

To assess de novo Cho synthesis, we utilized [14C] acetate. During the final 6 h of the 24-h treatment period, the culture medium was replaced with DMEM/F12 medium lacking both phenol red and SFB but containing [14C] acetate (3 PCi/ml in the final culture medium), either with or without 400 µM CuSO4. Following the treatment, astrocytes were rinsed with PBS (pH 7.4), detached from the plate via scraping, and then centrifuged at 500 g for 10 min. Subsequently, they were resuspended in 300 μL of lysis buffer (20 mM N-2-hydroxyethylpiperazine-N-2-ethanesulfonic acid (HEPES) at pH 7.40, 100 mM NaCl, 5 mM EDTA, and 1% v/v Triton X-100) and underwent sonication using a NUMAK LUZ-30A sonicator. Then, each sample was supplemented with 2 mL of hexane: isopropanol (3:2). The organic phase was meticulously retrieved using a glass Pasteur pipette, and the lipids were extracted following the Folch method [30], using Folch’s reagent (chloroform:methanol: water at a ratio of 2:1:0.8). After thorough mixing by vortexing, the samples were placed within ground glass tubes under a nitrogen atmosphere and allowed to stand overnight. Following saponification (with 10% potassium hydroxide for 1 h at 80 °C), the non-saponifiable fraction containing Cho was separated using high-resolution thin-layer chromatography (HP-TLC), with 100% chloroform as the mobile phase. A standard of Cho (Sigma, 57-88-5) was concurrently run, and detection was accomplished via autoradiography using a Storage Phosphor Screen from GE Healthcare. Plate development was conducted using Amersham’s Storm 860 Molecular Imager, and quantitative densitometric analysis was performed using Image J software. The total lipids concentration in each sample was determined by gravimetry [31]. Subsequently, the intensity of each band was normalized according to the concentration of lipids in the respective sample. The graphs were expressed as a percentage of the male control because no significant differences were observed between the male and female controls.

### Cho in lipid rafts

Neurons were harvested from the wells using 0.25% w/v trypsin, and trypsinization was halted by adding twice the volume of DMEM/F12 culture medium containing 10% FBS. The cells were then washed with cold PBS and suspended in a lysis buffer (20 mM HEPES at pH 7.40, 100 mM NaCl, 5 mM EDTA, and 1% v/v Triton X-100). The cell lysate was diluted with an equal volume of 90% (v/v) sucrose prepared in TNE buffer (10 mM Tris, 200 mM NaCl, and 1 mM EDTA at pH 7.4). Following the lysate containing 45% sucrose in TNE buffer, 2 mL of a solution comprising 35% sucrose in TNE buffer was layered, subsequently adding 1 mL of a solution containing 5% sucrose in TNE buffer. The samples were centrifuged at 190,000 g at 4 °C for 19 h using a Beckman SW60 Ti rotor, and 12 fractions of 0.33 mL each were collected. Lipids were isolated using the Folch method [30], and its concentration in each sample was determined by gravimetry [31] and resolved through HP-TLC as described earlier. The HP-TLC plate was immersed in a solution composed of 50 mg Cl_3_Fe + 90 mL H_2_O + 5 mL acetic acid + 5 mL SO_4_H_2_. Subsequently, the plate was heated in an oven at 100 °C for 3 min, resulting in the appearance of pink bands representing free Cho. The density of each band was analyzed using Image J. The intensity of each band was normalized according to the concentration of lipids in the respective sample.

### Western blotting

1X lysis buffer composed of 20 mM HEPES at pH 7.40, 100 mM NaCl, 5 mM EDTA, and 1% Triton X-100, along with 380 µL of a protease and phosphatase inhibitor cocktail (Thermo Scientific -78442), was added to each sample. The samples were then centrifuged at 10,000 rpm for 15 min at 4 °C, and the supernatant was collected. An equal concentration of protein per sample was separated using 15% polyacrylamide gels and subsequently transferred to polyvinylidene difluoride (PVDF) membranes (Immobilon™ transfer membranes, Millipore Corporation^®^) at 60 V for 1 h in a transfer buffer containing 20% (v/v) methanol in 48 mM Tris at pH 8.3- and 39-mM glycine. For immunodetection, the membranes were exposed to mouse monoclonal anti-APP antibody (SC-28365, Santa Cruz, CA, USA) diluted at 1/200, mouse monoclonal anti-β-actin antibody diluted at 1/3000 (Sigma A5316), goat polyclonal anti-ABCA1 antibody (SC-5491, Santa Cruz, CA, USA), and mouse monoclonal anti-Flotillin (sc-74566, Santa Cruz, CA, USA) diluted at 1/2000 for 1 h. Immunoreactive bands were visualized with SuperSignal™ West Pico PLUS Chemiluminescent Substrate (34577, Thermo Scientific) employing a ChemiDoc (ChemiDoc™ XRS, BIO RAD^®^) to capture fluorescence. The protein immunoblots were scanned, and the density of each band was quantified using ImageJ software (Image Processing and Analysis in Java). The graphs were expressed as a percentage of the male control because no significant differences were observed between the male and female controls.

Western blot samples were also used to assess intracellular Cu concentration, following established procedures [32]. In brief, the samples underwent acid digestion using a 3:1 mixture of nitric and hydrochloric acid. Subsequently, the Cu concentration was determined via Atomic Absorption Spectrometry (AAS) using the AAS 7000 spectrometer from Shimadzu, Japan, equipped with graphite furnace atomization (GF-AAS).

### RNA isolation and real-time PCR analysis

Samples were homogenized using Tripure isolation reagent (Roche Diagnostic, USA). The RNA extracted was subsequently reverse transcribed into complementary DNA (cDNA) using a commercial kit (1708891, Bio-Rad iScriptTM). The resulting cDNA was then amplified, employing Bio-Rad iQ SYBR Green Supermix (1708880, Bio-Rad). The quantitative polymerase chain reaction (qPCR) program comprised an initial step at 95 °C for 3 min, followed by 40 cycles (95 °C for 15 s and 60 °C for 60 s), and finally, a step at 95 °C for 1 min. Data analysis was carried out using the ΔΔCT method. The following oligonucleotide primers were employed:

**Table:** 

Gene	Forward primer	Reverse primer
SREBP-2	5ʹ-TCACTCCCTGGGAAAGT-3ʹ	5ʹ-CAGTAGCAGGCAGGCAGGCAGGCAGCAGGTCTCACAGGT-3ʹ
HMGCR	5ʹ-GGACTTCGAGCAAGAGAGAGAGAGAGAGAGAGAGAGAGATGGG-3ʹ	5ʹ-AGCACTGTGTTGCGTACAG-3ʹ
ß-actin	5ʹ-TCTTATTGGTCGAAGGCTCGT-3ʹ	5ʹ-ATCTCACTAGAGGCCACCGA-3ʹ

### Protein determination

We employed the Bradford method to ascertain the protein content in the samples [33].

### Statistical analyses

All the values represent the mean ± SD (standard deviation) of independent determinations. Data were analyzed first by the Shapiro–Wilk normality test and then by ANOVA or 2-way ANOVA followed by the corresponding multiple comparison test using GraphPad Prism 6 software.

## Results

### Determination of sublethal Cu concentration

**Fig. 1:**
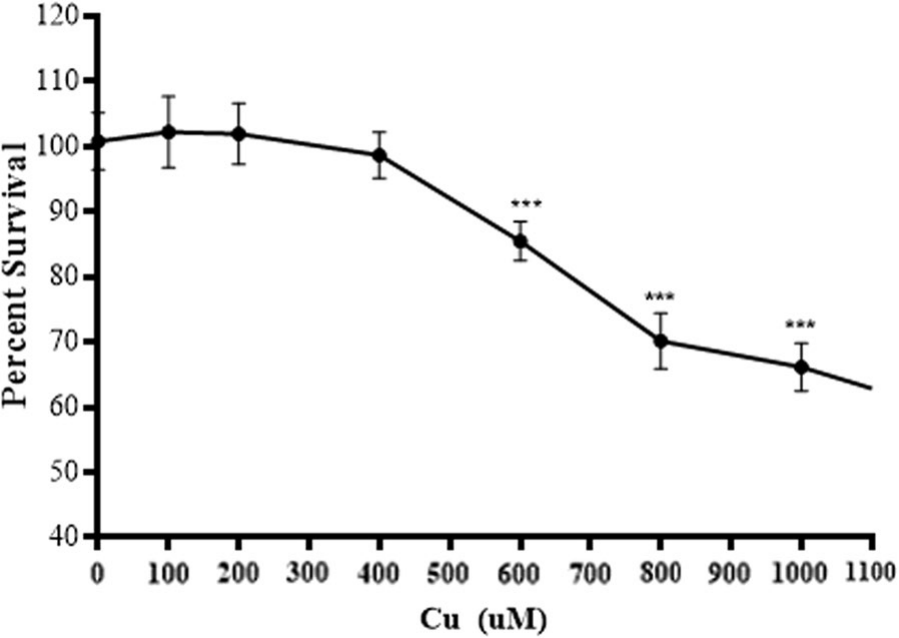
Cell viability. Astrocytes were cultured and treated for 24 h with increasing concentrations of CuSO_4_. Cell viability was determined by resazurin assay. Results were calculated using ANOVA and Bonferroni’s multiple comparisons test, and data expressed mean ± SD percentage of control (n = 6 for each concentration used). Statistical differences are indicated as ***p < 0.01

To establish the optimal Cu concentration for our experiments, we conducted a survival curve analysis to identify the highest Cu concentrations that did not have a detrimental effect on cell viability, thereby maintaining a sublethal condition (Fig. 1). To achieve this, astrocytes were exposed to various Cu concentrations for 24 h. We found that 400 µM Cu was the highest concentration at which no cell mortality was observed, and we therefore chose this concentration for subsequent experiments (Fig. 1).

### Cu and oxidative stress

**Fig. 2:**
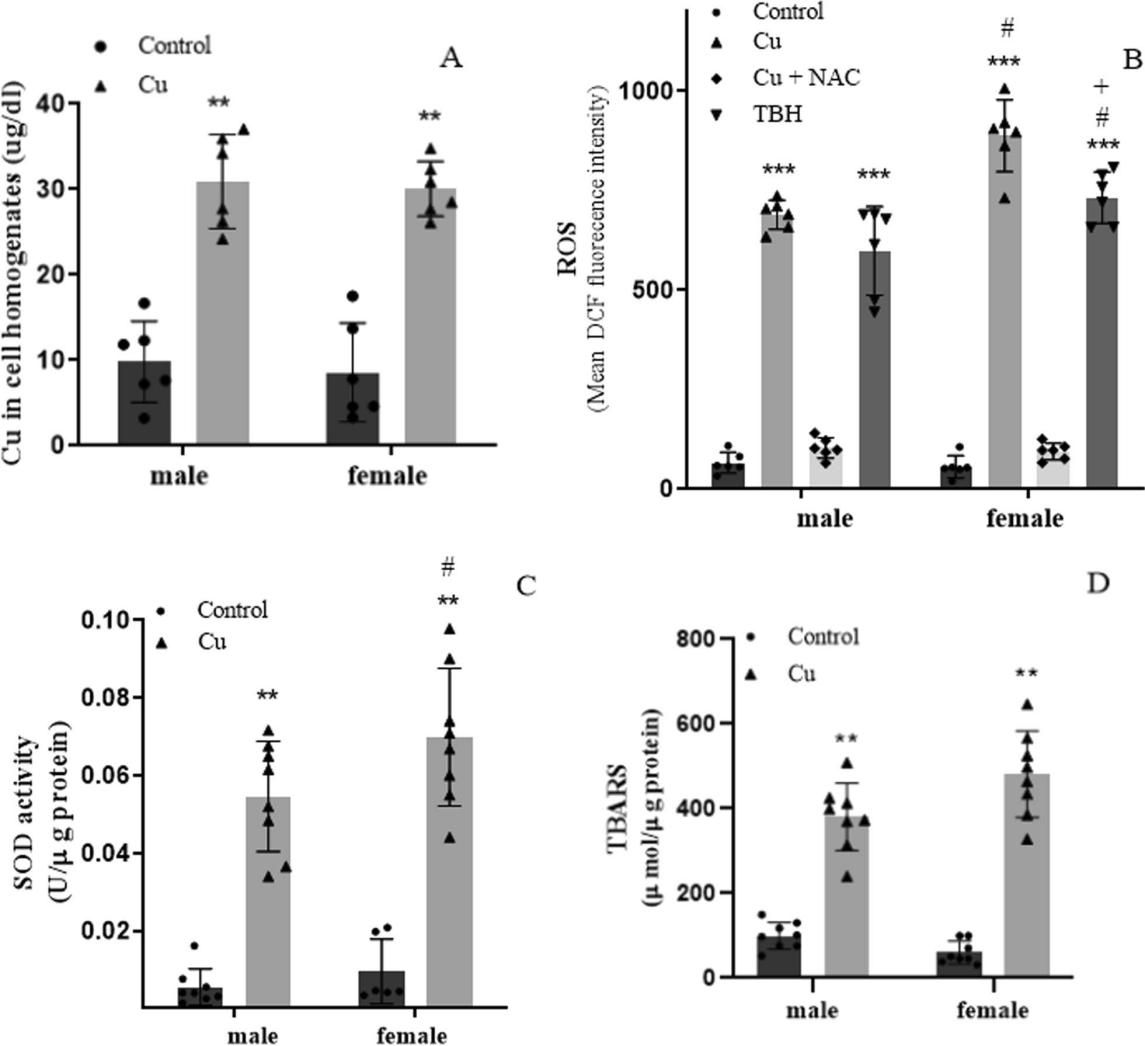
Cu treatment effect on oxidative stress in female and male astrocytes and cellular Cu content. **A** Cellular Cu concentration (µg/dL) measured by spectrophotometry (n = 6); **B** ROS levels were determined using DCF-DA by flow cytometry (n = 6); **C** SOD activity was measured by spectrophotometry (n = 6 to 8); and **D** Lipid peroxidation was determined by the TBARS assay (n = 8). All determinations were made after 24 h of Cu treatment (400 µM of CuSO_4_; gray bar). Untreated cells were used as controls (black bar), TBH (500 µM) was used as a positive control of ROS production (dark gray bars), and NAC (10 µg/mL) was used as an antioxidant molecule to counteract Cu ROS generation to be used as a second negative control (light gray bars). Results were calculated using 2-way ANOVA plus Bonferroni’s multiple comparisons test, and data expressed mean ± SD, **p < 0.01 and ***p < 0.001 significant differences respect to the control of the same sex, ^#^significant differences between female and male astrocytes with the same treatment (p < 0.05 for TBH,  p < 0.01 for Cu ROS and p < 0.1 for Cu SOD activity), and + p < 0.05 significant differences between Cu and Cu + NAC treatment of the same sex

To explore the potential cytotoxic effects arising from sublethal Cu overload on both female and male astrocytes, we initially aimed to ascertain the extent of Cu entry into these cells. We found that astrocytes, irrespective of their gender of origin, showed similar levels of Cu uptake (Fig. 2A). We then assessed ROS production via flow cytometry employing DCF-DA after treating the cells for 24 h with 400 µM Cu. Interestingly, our results demonstrated that Cu and TBH exposure induced significantly greater ROS generation in female than in male astrocytes (with p-values of < 0.01 and < 0.05, respectively), as depicted in Fig. 2B. Matching the ROS observations, SOD activity mirrored these trends (as shown in Fig. 2C). However, while Cu treatment led to an increase in TBARS levels, no statistically significant differences were discerned between male and female astrocytes in lipid peroxidation (Fig. 2D).

### Cu effects on Cho de novo synthesis

**Fig. 3:**
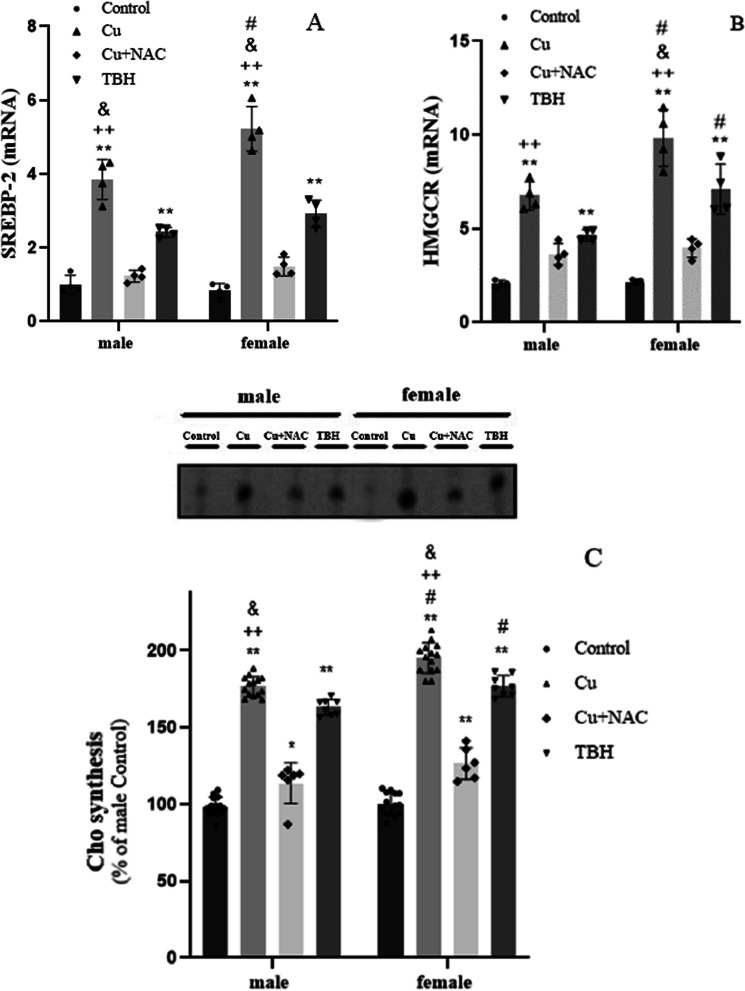
Cu effect on Cho de novo synthesis. **A** Expression levels of *SREBP-2* determined by RT-qPCR (n = 4); **B** Expression levels of *HMGCR* determined by RT-qPCR (n = 4); **C** Cho de novo synthesis determined by HP-TLC followed by the intensity analysis of radioactive bands (n = 6–14). All determinations were made after 24 h of Cu treatment (400 µM of CuSO_4_; gray bar). Untreated cells were used as controls (black bar), TBH (500 µM) was used as a positive control of ROS production (dark gray bars), and NAC (10 µg/mL) was used as an antioxidant molecule to counteract Cu ROS generation to be used as a second negative control (light gray bars). Results were calculated using 2-way ANOVA plus Bonferroni’s multiple comparisons test, and data expressed mean ± SD, *p < 0.05 and **p < 0.01 significant differences respect to the control of the same sex, and ^#^ significant differences between female vs male astrocytes with same treatment (p < 0.05 for SREBP-2, p < 0.01 for Cu HMGCR, p < 0.1 TBH HMGCR, p < 0.01 for Cu Cho de novo synthesis and p < 0.05 for TBH Cho de novo synthesis), + + p < 0.01 significant differences between Cu and Cu + NAC treatment of the same sex, and & p < 0.01 significant differences between Cu and TBH treatment of the same sex

Our findings indicate that Cu treatment significantly increased the expression of SREBP-2 in astrocytes, with a higher effect in female than in male astrocytes (p < 0.01 and p < 0.05, respectively) (Fig. 3A). Notably, similar upregulation of SREBP-2 expression was observed with TBH treatment, but this effect was not seen with Cu + NAC treatment. This suggests that Cu and TBH induce this increase through the generation of reactive oxygen species (ROS). Consistent with the SREBP-2 findings, we observed an increase in HMGCR expression after 24 h of Cu and TBH treatment, with female astrocytes showing more pronounced increases than male astrocytes (Fig. 3B). It is worth noting that Cu + NAC treatment significantly increased HMGCR expression only in female cells (Fig. 3B). Furthermore, our study revealed that Cu and TBH treatments led to a notable increase in de novo Cho synthesis in primary astrocyte cultures, with a more pronounced effect in female astrocytes (p < 0.01), as seen in Fig. 3C. However, Cu treatment resulted in a more significant increase in Cho synthesis than TBH. Interestingly, when astrocytes were treated with Cu + NAC, the levels of de novo synthesized Cho were significantly higher in both male and female astrocytes. However, these levels were still significantly lower than in treatment with Cu or TBH alone. This underscores the intricate relationship between Cu, NAC, and Cho synthesis.

### Cho release

**Fig. 4:**
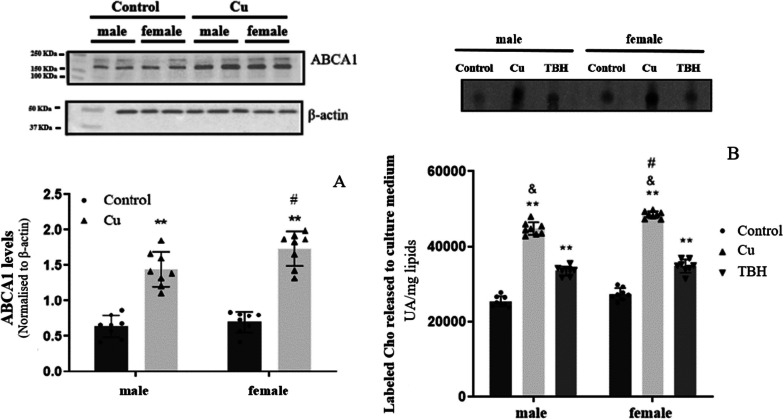
Effects of Cu on Cho release. **A** ABCA1 levels determined by western blot (n = 8); **B** Labeled Cho (^14^Cho) produced by de novo synthesis determined by HP-TLC followed by the analysis of intensity of radioactive bands (n = 8). All determinations were made after 24 h of Cu treatment (400 µM of CuSO_4_; gray bar). Untreated cells were used as controls (black bar), TBH (500 µM) was used as a positive control of ROS production (dark gray bars), and NAC (10 µg/mL) was used as an antioxidant molecule to counteract Cu ROS generation to be used as a second negative control (light gray bars). Results were calculated using 2-way ANOVA plus Bonferroni’s multiple comparisons test, and data expressed mean ± SD, **p < 0.01 significant differences respect to the control of the same sex, ^#^ significant differences between Cu female vs Cu male (p < 0.05 for ABCA1 and p < 0.01 for ^14^Cho release), and ^&^p < 0.01 significant differences between Cu and THB treatments in the same sex

To investigate the potential release of de novo synthesized Cho into the extracellular medium due to Cu treatment, we assessed both the protein levels of the Cho transporter, ABCA1, in cell homogenates (Fig. 4A) and the levels of labeled Cho in the culture medium (Fig. 4B). Following Cu treatment, we observed higher ABCA1 levels, with this increase being more prominent in female than in male astrocytes (p < 0.01) (Fig. 4A). Similarly, we also detected an increase in de novo synthesized Cho released into the culture medium, which paralleled the behavior of ABCA1 (p < 0.01). Notably, it is interesting that Cu treatment, whether applied to male or female astrocytes, resulted in a higher release of labeled Cho into the culture medium compared to TBH treatment (p < 0.01) (Fig. 4B).

### Effect of astrocyte-derived conditioned culture medium on Cho and APP levels in neuronal membrane rafts

**Fig. 5:**
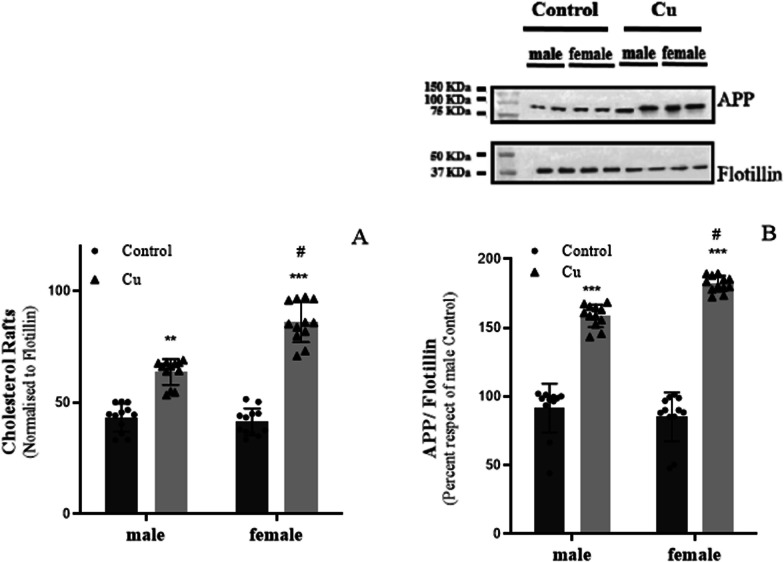
Effect of astrocyte-conditioned culture medium on Cho and APP levels in neuronal membrane rafts. **A** Cho levels in membrane rafts of neurons determined by HP-TLC followed by the analysis of intensity of bands (n = 12). **B** APP/flotillin ratio in neuronal membrane rafts determined by western blot (n = 12). All determinations were made after 24 h of neuronal primary culture exposure to conditioned astrocyte culture medium previously exposed or not (control; black bars) to 400 µM of CuSO_4_ (gray bar). Results were calculated using 2-way ANOVA plus Bonferroni’s multiple comparisons test, and data expressed mean ± SD, **p < 0.01 and ***p < 0.001 significant differences between Cu vs control, ^#^p < 0.01 significant differences between Cu female vs Cu male

Given our observation of an elevated efflux of labeled Cho into the culture medium, and the established knowledge that mature neurons incorporate Cho synthesized by astrocytes, thereby impacting Cho concentration in membrane rafts [3], we embarked on an analysis of Cho levels within neuronal membrane rafts. For this purpose, we treated neurons in primary cultures to conditioned culture mediums obtained from astrocytes exposed to sublethal Cu overload for 24 h. We found that neuronal Cho levels within membrane rafts were notably higher in neurons exposed to the conditioned medium from Cu-treated astrocytes than in those exposed to the conditioned control medium (Fig. 5A). Additionally, these Cho levels were even more elevated in neurons exposed to the conditioned culture medium derived from female Cu-exposed astrocytes than to that from Cu-exposed astrocytes (p < 0.01). Since it is known that in the mature brain, neurons meet their Cho requirements mainly by acquiring that which is synthesized by astrocytes [3, 7], we infer that the heightened Cho levels are likely a consequence of Cho uptake from the culture medium. Considering the influence of Cho concentrations within lipid rafts on the transport of APP into or out of these rafts, we then investigated the levels of this protein within neuronal rafts. In line with the Cho levels observed within the rafts, the most significant increase in APP levels was in neurons exposed to the conditioned medium derived from Cu-exposed female astrocytes, followed by those exposed to medium from Cu-exposed male astrocytes (p < 0.01) (Fig. 5B).

## Discussion

As mentioned, AD is the most common cause of dementia worldwide, and yet there is no cure, no diagnosis, and no preventive treatment before the onset of clinical symptoms. The Alzheimer’s Association also points out that AD appears to affect more women than men [17], although the reason for this is still unknown. Several studies demonstrated that brain Cho concentrations are higher in people suffering from AD than in healthy adults [34, 35]. In neurons, an excess of Cho leads to an increased distribution of APP within membrane rafts. This subsequently results in higher levels of Aβ and phospho-Tau, which can trigger memory deficits and lead to hippocampal atrophy, and may contribute to the onset of AD [36].

We and others demonstrated that people can be exposed to Cu overload in different ways [12, 13, 37]. It has been observed that individuals with AD exhibit elevated levels of Cu in plasma [9, 38]. In this context, we recently showed that a sublethal concentration of Cu can induce Cho de novo synthesis in immature neurons [16]. Given that astrocytes play a pivotal role in the synthesis and supply of Cho to neurons within the mature brain, our objective was to investigate the potential sex-dependent effects of sublethal Cu concentrations on Cho metabolism in primary astrocyte cultures. Additionally, we aimed to examine any potential associations between these effects and variations in Cho levels within neuronal lipid rafts, as well as alterations in APP levels.

First, the intracellular concentration of Cu was analyzed after 24 h of treatment. As expected, astrocytes exposed to 400 µM Cu had significantly higher intracellular Cu concentrations than the control group, but no significant differences were observed between male and female astrocytes. Since Cu is a redox-active transition metal that can participate in Fenton or Haber–Weiss reactions that increase ROS [39], it was not surprising to observe an increase in ROS production after Cu treatment. Interestingly, female astrocytes exhibited a significantly higher production of ROS than male astrocytes, not only in response to Cu treatment but also when exposed to TBH. Thus, it appears that female astrocytes are more sensitive to ROS-generating substances. While we cannot definitively ascertain the reason why female astrocytes seem more sensitive to ROS-generating agents, we can rule out the possibility of it being related to a differential level of maturation, as previously observed in various brain regions in postnatal rat astrocytes [40]. This is supported by the fact that both male and female astrocytes obtained from rats displayed equivalent levels of the glial fibrillary acidic protein (GFAP) (data not shown), which serves as a marker for astrocytic maturation [40]. However, sex differences in astrocytes can arise from differential exposure to sex steroids during development. It is known that testosterone peaks occur in males on the 18th day of gestation and at birth, whereas in females, the hormonal peak occurs only during puberty [41, 42]. Regarding males, the peak of estradiol derived from testicular testosterone is crucial for the sexual differentiation of the male brain, and it induces sex differences in CpG methylation [43]. A translatome analysis indicates that cortical astrocytes derived from males show an earlier mature phenotype than females. Moreover, female astrocytes exhibit reduced expression of certain demethylases, suggesting the possibility of lower activity in these enzymes [44]. The differential methylation patterns between male and female astrocytes could modify the expression of enzymes involved in the antioxidant response. In this regard, it could be proposed that there might be a sex-related antioxidant defense response, regardless of SOD activity, that could contribute to a more effective defense mechanism in males against similar increases in stressors, such as Cu or TBH, resulting in lower levels of ROS. Additionally, in vitro observations demonstrate that testosterone treatment increases the activity of SOD and also another antioxidant enzyme, catalase, in response to ROS induction [45]. It is crucial to specify that all these ideas are theoretical conjectures, which would be extremely interesting to investigate in future studies. Notably, despite the heightened SOD activity and TBARS levels resulting from Cu treatment, no disparities in astrocyte viability were noted when compared to control cells.

Several works have shown that ROS can not only oxidize molecules but also act as second messengers [46, 47]. In fact, ROS can increase in vitro the expression of the transcription factor *SREBP-2*, responsible for promoting the transcription of genes related to Cho metabolism [48]. Consistent with previous research, our study revealed an elevation in SREBP-2 expression following treatments that generate ROS (Cu and TBH). Notably, despite both treatments generating similar levels of ROS, Cu induced a greater upregulation of SREBP-2 than TBH, and this induction was even more pronounced in female astrocytes. Remarkably, SREBP-2 levels exhibited no statistically significant alteration when Cu was co-administered with the antioxidant molecule NAC. Consequently, in agreement with previous studies [46, 49], we infer that the rise in SREBP-2 is likely primarily attributable to ROS. As we mentioned previously, SREBP-2 is a transcription factor that promotes the expression of genes related to Cho synthesis, such as HMGCR (the main regulatory enzyme of the mevalonate pathway) [50, 51]. In agreement with previous works, the elevation in SREBP-2 coincided with the increase in HMGCR expression [49, 52], and this elevation was once again more pronounced in female than in male astrocytes. Notably, we observed that following Cu + NAC treatment, which did not lead to a substantial increase in SREBP-2 expression and ROS levels compared to the control group, there was a marked upregulation in HMGCR expression in female astrocytes. While male astrocytes exhibited a similar trend toward increase, it did not reach statistical significance. Thus, we demonstrated for the first time that it is possible for Cu to directly affect HMGCR expression.

Consistently, the rise in HMGCR expression was accompanied by an increase in de novo Cho synthesis. The de novo Cho levels mirrored the patterns observed with HMGCR, showing an increase after Cu and TBH treatment, as well as after Cu + NAC treatment in both male and female astrocytes. The fact that more Cho was synthesized after Cu + NAC treatment, while we observed no increases in ROS compared to the control, suggests that this metal may be directly influencing Cho synthesis, in addition to its role in ROS generation. In line with this, we recently demonstrated in vitro that Cu, unlike iron or zinc, may promote de novo Cho synthesis in a ROS-independent manner [16].

Multiple studies have shown that Cho synthesized by astrocytes is primarily integrated into lipoproteins for secretion into the extracellular medium through specific transporters, and subsequently taken up by neurons [2, 5]. Within astrocytes, ABCA1 plays a crucial role in promoting the efflux of Cho to lipid-free apo A-I, contributing to the biogenesis of lipoproteins [53]. In line with this, a reduction in Cho release from astrocytes has been associated with a concurrent decrease in ABCA1 levels [54]. In the present study, ABCA1 levels increased following Cu treatment and this increase was more pronounced in female astrocytes than in males. Since the activation of Cho synthesis (via the mevalonate pathway) is necessary for the formation of oxysterols that promote the activation of the LXR (liver X receptor) receptor [55, 56], which in turn regulates the transcription of ABCA1 [55], we can speculate that the increase in ABCA1 may be related to the increase observed in Cho synthesis. This may also explain the greater elevation of ABCA1 levels in female astrocytes compared to males.

All these results become even more significant when considering that adult neurons primarily incorporate Cho synthesized by astrocytes (astrocytes are responsible for its production and transport to neurons [2, 4]) and that astrocyte-derived Cho modulates APP trafficking into and out of membrane rafts [7]. APP amyloidogenic processing leads to Aβ formation which eventually develops amyloidogenic plaque. An increased concentration of astrocyte-derived Cho in neurons has been shown to increase Cho in membranes leading to a rise in the size (but not in number) of lipid rafts and of APP within rafts, favoring the amyloidogenic processing pathway [7]. In line with this, we showed higher levels of Cho and APP in neuronal rafts that have been treated with the conditioned culture medium derived from astrocytes treated with Cu. Finally, even more interestingly, female neurons exposed to the culture medium of female astrocytes treated with Cu, which showed significantly higher levels of labeled Cho than male neurons under the same treatment, also exhibited more Cho and APP in membrane rafts.

### Perspectives and significance

In summary, this study demonstrates that Cu promotes Cho synthesis in astrocytes. This increase in Cho synthesis appears to be ROS-dependent, as well as ROS-independent, as we have observed previously [16]. It is worth noting that female astrocytes exhibited higher levels of HMGCR and de novo Cho synthesis than males under the same treatments (Cu and TBH), indicating that they may be more susceptible to ROS-generating agents. In this regard, there is in vivo evidence demonstrating that, despite similar levels of Aβ in the brains of female and male mice, females exhibited greater memory loss, suggesting that females may be more susceptible to the toxic effects of Aβ than males [57].

The most significant increase in Cho exported to the culture medium observed in female astrocytes matched the highest elevation of Cho and APP in neuronal rafts. Since we previously observed in vitro that increases in APP levels within the rafts are associated with increases in Aβ [16], we hypothesized that the increase in APP in neuronal rats could also be linked to an elevation of this peptide. Therefore, we speculate that the elevated risk of AD in females may result not solely from metabolic alterations triggered by sex hormones or the disparity in lifespan between genders [21]. It could also stem from gender-specific variations in the response to metals or external substances, affecting the regulation of key enzymes involved in diverse biochemical pathways, such as HMGCR. We consider that this study, which highlights a sex-dependent response in the de novo Cho synthesis of astrocytes exposed to ROS-generating agents like TBH and, especially, Cu, and its impact on neuronal raft APP levels, could stimulate further research aimed at uncovering the underlying causes of the disproportionate prevalence and severity of AD in women compared to men.

## Data Availability

The datasets during and/or analyzed during the current study are available from the corresponding author on reasonable request.
